# Transcriptomic Studies of Malaria: a Paradigm for Investigation of Systemic Host-Pathogen Interactions

**DOI:** 10.1128/MMBR.00071-17

**Published:** 2018-04-25

**Authors:** Hyun Jae Lee, Athina Georgiadou, Thomas D. Otto, Michael Levin, Lachlan J. Coin, David J. Conway, Aubrey J. Cunnington

**Affiliations:** a Institute for Molecular Bioscience, University of Queensland, Brisbane, Australia; b Section of Paediatrics, Imperial College, London, United Kingdom; c Centre of Immunobiology, University of Glasgow, Glasgow, United Kingdom; d Department of Pathogen Molecular Biology, London School of Hygiene and Tropical Medicine, London, United Kingdom

**Keywords:** RNA sequencing, transcriptomics, apicomplexan parasites, host-parasite relationship, host-pathogen interactions, immune response, malaria, pathogenesis

## Abstract

Transcriptomics, the analysis of genome-wide RNA expression, is a common approach to investigate host and pathogen processes in infectious diseases. Technical and bioinformatic advances have permitted increasingly thorough analyses of the association of RNA expression with fundamental biology, immunity, pathogenesis, diagnosis, and prognosis. Transcriptomic approaches can now be used to realize a previously unattainable goal, the simultaneous study of RNA expression in host and pathogen, in order to better understand their interactions. This exciting prospect is not without challenges, especially as focus moves from interactions *in vitro* under tightly controlled conditions to tissue- and systems-level interactions in animal models and natural and experimental infections in humans. Here we review the contribution of transcriptomic studies to the understanding of malaria, a parasitic disease which has exerted a major influence on human evolution and continues to cause a huge global burden of disease. We consider malaria a paradigm for the transcriptomic assessment of systemic host-pathogen interactions in humans, because much of the direct host-pathogen interaction occurs within the blood, a readily sampled compartment of the body. We illustrate lessons learned from transcriptomic studies of malaria and how these lessons may guide studies of host-pathogen interactions in other infectious diseases. We propose that the potential of transcriptomic studies to improve the understanding of malaria as a disease remains partly untapped because of limitations in study design rather than as a consequence of technological constraints. Further advances will require the integration of transcriptomic data with analytical approaches from other scientific disciplines, including epidemiology and mathematical modeling.

## INTRODUCTION

Transcriptomics is the quantitative or qualitative study of RNAs on a genome-wide scale (1). It is just one of several powerful approaches to undertake comprehensive or global analyses of large sets of related features, such as genes (genomics), proteins (proteomics), DNA modifications (epigenomics), or microbial communities (microbiomics). These approaches are often used for discovery rather than hypothesis-based investigation, since they can provide an unbiased description of similarities or differences between conditions of interest. The development of technologies for these high-dimensional analyses has been accompanied by novel computational and analytic approaches to deal with the vast amounts of data and has driven the emergence of the scientific discipline of bioinformatics (2).

Initially, transcriptomic studies sought to quantify the expression levels of protein-encoding genes, often with the implicit assumption that this would broadly indicate changes in protein expression levels (3). However, as technologies and the understanding of noncoding RNAs have evolved, transcriptomic approaches have allowed a much deeper understanding of the complexities of the regulation of gene expression, alternate splicing events, and functions of noncoding RNAs as well as proving invaluable for the accurate construction and annotation of complex genomes (4–7). Combinations of genomic, epigenomic, transcriptomic, and proteomic approaches are now increasingly being applied to provide a deeper understanding of the multiple layers of control that result in variations between cells, tissues, individuals, and populations in either health or disease (1, 7–10).

### Malaria

Malaria is a parasitic disease caused by apicomplexan parasites of the genus Plasmodium, which can infect a diverse range of vertebrate hosts. A comprehensive description of malaria epidemiology, biology, immunology, pathogenesis, and treatment is beyond the scope of this text and has been covered in recent review articles (11–16). Here we give a brief overview, and additional background accompanies relevant sections below in this article.

Five main species of Plasmodium cause most disease in humans: P. falciparum, P. vivax, P. knowlesi, P. malariae, and P. ovale. P. falciparum is the major cause of severe malaria, which can result in death, and is the focus of most of the human studies discussed in this article. The term malaria refers to the disease caused by infection with these parasites, and individuals with asexual-stage parasites in their blood without symptoms are described as having asymptomatic parasitemia (11).

**FIG 1:**
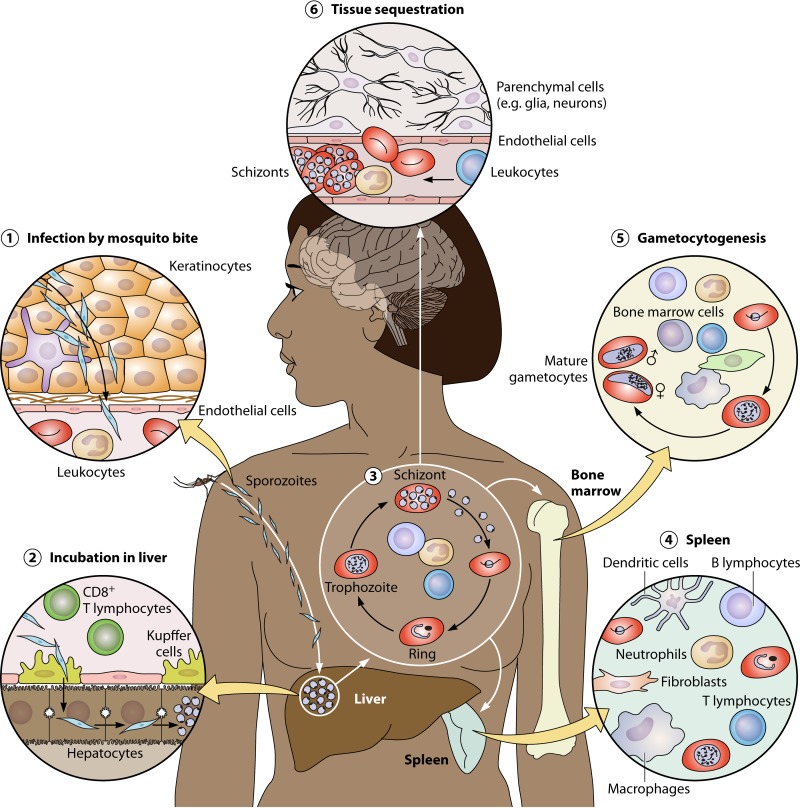
Main interactions with human tissues and cells during the Plasmodium falciparum developmental cycle. (1) Infection is initiated by a mosquito bite. Motile sporozoites rapidly find their way past the structural and immune cells in the skin into blood vessels and onwards to the liver. The short transit time limits opportunities for cellular interactions. (2) Sporozoites reach the liver, exit the vasculature through Kupffer cells, and then undergo massive replication in hepatocytes. Immune cells such as CD8 T cells patrol the liver and may detect and kill infected hepatocytes. (3) Parasites burst out of hepatocytes and enter the bloodstream, rapidly infecting erythrocytes. They undergo repeated cycles of asexual replication, interacting with blood leukocytes. Parasite products are carried throughout the systemic circulation, triggering inflammatory responses. (4) Parasitized red cells may be cleared by the spleen, which is a major location for the host immune response to Plasmodium. (5) Parasites may exit the asexual erythrocytic cycle to produce gametocytes, which may be taken up by another mosquito bite to allow onward transmission. Gametocytogenesis may be influenced by the host response, and most gametocyte development occurs in the bone marrow. Mature gametocytes reenter and circulate in the blood, potentially interacting with host leukocytes and the vascular endothelium. (6) Parasites can cause severe disease if they accumulate (sequester) and obstruct the microvasculature of vital organs, such as the brain. There may be both direct and indirect interactions with the vascular endothelium, leukocytes, and parenchymal cells.

Plasmodium species are transmitted to humans by the bite of female Anopheles mosquitoes, and motile forms of the parasite (sporozoites) quickly make their way from the skin to blood vessels to hepatocytes, where they undergo massive intracellular asexual replication during the incubation phase of infection (Fig. 1). After one or more weeks, the brood of parasites escapes from the hepatocyte and reenters the bloodstream, but this time, the parasites rapidly invade red blood cells (RBCs), where they undergo repeated asexual reproductive cycles, with new parasites being produced every 24 to 72 h, depending on parasite species (11).

For P. falciparum, around 16 to 32 daughter parasites are released approximately every 48 h. Parasite numbers increase exponentially until they are sufficient to trigger a host response, which starts to constrain this growth and also results in symptoms such as fever, muscle aches, headache, and violent shivering (rigors) (11–13). Often, symptoms occur in paroxysms, coinciding with the rupture of RBCs and the release of new parasites and their pathogen-associated molecular patterns into the circulation, triggering responses through host pattern recognition receptors (16).

If parasite numbers continue to increase, because of insufficient constraint by the host response or a failure to receive antimalaria treatment, this predisposes an individual to an increasing risk of severe disease manifestations, which may include coma, lung injury, renal failure, acidosis, and severe anemia (11, 13, 16). These manifestations are thought to be consequences of not only high parasite loads but also high levels of inflammation; dysfunction of the vascular endothelium, which impairs its ability to regulate blood flow and prevent coagulation; and obstruction of small blood vessels by adherent parasitized erythrocytes (sequestration) (12, 13, 15, 16).

Indeed, extensive sequestration is a unique feature of P. falciparum and is largely explained by specific adhesive interactions between vascular endothelial surface molecules and parasite molecules expressed on the surface of infected RBCs (iRBCs) (17–19). However, the parasite will not benefit if inexorable growth kills its host too quickly, and at some stage in the intraerythrocytic asexual replication cycle, a proportion of parasites begins to differentiate into sexual stages (gametocytes), which can be taken up in the blood meal of another mosquito (20). The sexual phase of the life cycle then occurs in the mosquito gut, with a brief diploid stage before new haploid parasites eventually make their way to the salivary glands as sporozoites, ready to infect a new human host during a future blood meal (15).

Individuals in some areas where malaria is highly endemic will be exposed to multiple infectious mosquito bites every day, and cumulative infections result in the acquisition of clinical immunity. First, individuals cease to be vulnerable to severe disease, and they then become less likely to develop clinical symptoms (12). This clinical immunity is thought to be largely antibody mediated and may reflect the ability to reduce, but not completely prevent, parasite replication through the acquisition of an increasing breadth of antibodies against polymorphic parasite antigens (14, 15, 21). Naturally acquired sterile immunity is thought to be very rare (perhaps never occurring), and the need to considerably improve on this natural immune response to highly antigenically diverse parasites has also produced challenges for vaccine development (14, 15).

### Malaria as a Paradigm for Transcriptomic Studies of Systemic Host-Pathogen Interactions

Clinical manifestations of malaria are due to the asexual blood stage of the parasite, and the host-parasite interactions that cause disease occur within the vasculature and its contiguous organs, such as the spleen (13, 16, 22). This means that many aspects of the host-pathogen interaction can be assessed through analyses of circulating blood. Whole blood (containing leukocytes and RBCs) can be used as a source of both host and parasite cells, and transcriptomic analyses can be applied to examine either cell type (23, 24) or both cell types in the same sample (25). The high numbers of parasites that can be found in human blood, particularly in children with some forms of severe malaria (26, 27), can yield abundant parasite RNA, making this a feasible approach. Furthermore, the pathogen load can be estimated from an examination of blood for parasites or their products (18, 26–29). In our experience, parasitemia levels as low as 2% can yield sufficient parasite RNA reads for meaningful analysis with standard-depth RNA sequencing (RNA-seq) (30 million to 40 million reads), but lower parasitemia levels may require greater sequencing depth.

The pathogen load is likely to be an important factor in determining the pathogenesis of many infectious diseases but is much harder to quantify as a stimulus for the systemic host response when pathogens are differentially distributed throughout multiple tissues (30). On a reductionist scale, transcriptomic studies of bacterial infections in cell culture models have identified reciprocal interactions between host and pathogen that contribute to pathogen growth (31, 32), but understanding how this relates to disease requires evaluation at a much larger scale. Evaluation of host-parasite interactions in blood in malaria through transcriptomic analyses provides a paradigm for understanding the role of systemic host-pathogen interactions in general.

### Aims and Scope of This Review

Here we aim to describe the contribution that transcriptomic studies have already made to understanding malaria and highlight how new approaches might permit greater insights. We provide an introduction to existing and new technologies and analytical approaches and outline how these technologies are transforming the depth and richness of transcriptomic data. We then consider malaria as a paradigm for transcriptional analyses of systemic host-pathogen interactions, lessons learned from malaria studies, and strategies for application to other infectious diseases. We illustrate that the traditional approach of considering variation in the host response to an invariant pathogen is too simplistic and that accumulating evidence suggests that dynamic variation in pathogen behavior also needs to be considered.

## TRANSCRIPTOMIC APPROACHES TO HOST-PATHOGEN INTERACTIONS

**FIG 2:**
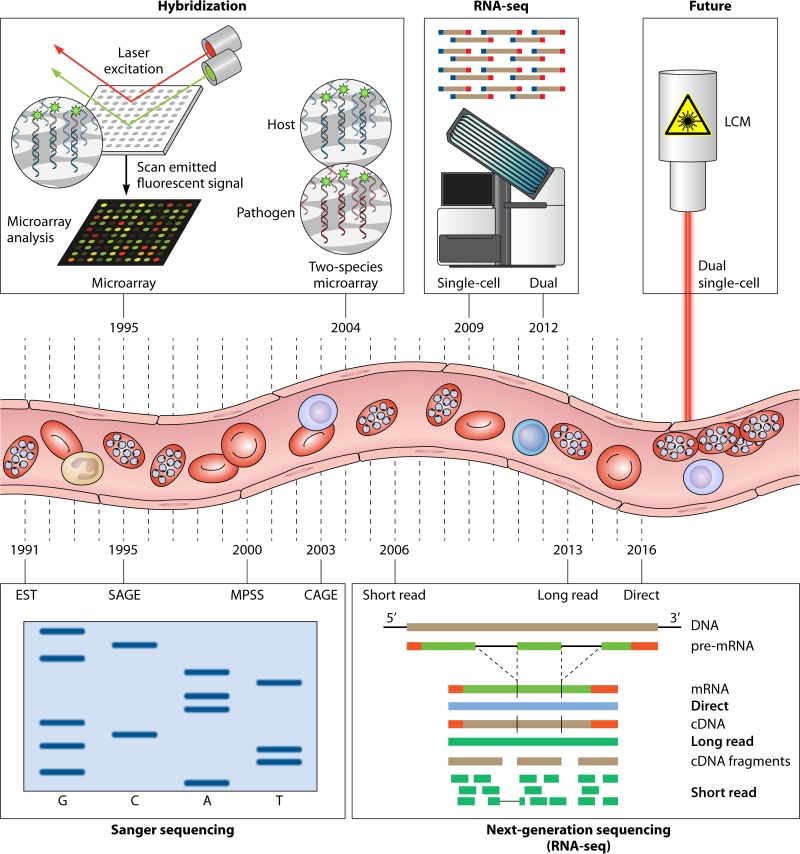
Timeline of transcriptomic approaches for infectious diseases. Transcriptomic analysis requires the extraction of RNA from parts of the body such as peripheral blood. Methods of analysis have evolved over time. Serial analysis of gene expression (SAGE) utilizes the Sanger sequencing approach to generate and sequence short (∼11-nucleotide) tags and quantify transcript abundance. It is expensive and low throughput. Massively parallel signature sequencing (MPSS) generates slightly longer tags (∼17 to 20 nucleotides) and provides a larger library size. Cap analysis of gene expression (CAGE) is similar in principle to SAGE but targets transcription start sites. Microarray analysis is a hybridization approach that uses fluorescence-tagged probes to target transcripts of interest. RNA sequencing (RNA-seq) is a high-throughput sequencing approach capable of novel transcript discovery, noncoding RNA analysis, and alternative splicing analysis. RNA-seq has been developed to allow transcriptomic analysis at a single-cell resolution, simultaneous analysis of host and pathogen transcriptomes (dual RNA-seq), and sequencing of full-length transcripts to allow detailed analysis of transcript isoforms and direct analysis of RNA. In the future, techniques such as laser capture microdissection (LCM) may be coupled with RNA-seq to allow host cells and their interacting pathogens (such as parasites adhering to vascular endothelial cells) to be isolated and studied as defined cell groups or dual single-cell analyses. Massively parallel single-cell analyses and direct RNA-seq are also on the horizon.

There are numerous excellent reviews of transcriptomic technologies, including comparisons of their relative merits and detailed methodological considerations (4, 33–37), so we highlight only selected characteristics here. The development of these technologies over the last 2 decades (Fig. 2) has driven many transcriptomic studies of malaria and other infectious diseases, sometimes with more emphasis on applying new technology than on addressing important biological and clinical questions. Microarray technologies were initially the only commercial transcriptomic tools, but they have never been ideally suited to studies of host-pathogen interactions because of the limited range of species covered by commercial arrays and the major restriction that probes must be designed for known or predicted transcripts. Thus, initial microarray studies focused largely on host gene expression, and as pathogen genomes were assembled, pathogen gene expression analyses then became possible. Eventually, attempts were made to study both host and pathogen together by using custom-made arrays, but these methods never achieved great popularity because of design challenges. The inherent limitations of microarray technology (Table 1) mean that it is being superseded by RNA-seq as the preferred approach for many transcriptomic applications, including studies of infectious diseases.

**TABLE 1:** Comparison of microarray and RNA sequencing technologies

Feature	Description		Reference(s)
	Microarray	RNA sequencing	
Technology	Tens of thousands of DNA probes specific for each transcript of interest within microwells on a chip; capture of cDNA produces a fluorescent signal proportional to target abundance	Next-generation sequencing of many millions of cDNA fragments	4, 38
Reference genome dependent	Yes; probes must be designed for specific targets	No; *de novo* assembly of transcripts is possible, reference is helpful for quantification	4, 38
Sample requirement(s)	Good-quality, nondegraded RNA	Good-quality, nondegraded RNA but possible with small amounts and partially degraded samples; rRNA depletion is usually required	37, 39
Dynamic range for transcript detection	Fixed	Unlimited but influenced by sequencing read depth	40
Novel transcript detection	No	Yes	41, 42
Noncoding RNA detection	Yes	Yes	41–43
Splice detection	Yes but limited to known splice sites	Yes	44, 45
Dual host-pathogen transcript detection	Yes but requires custom arrays designed for both transcriptomes	Yes; relative amounts of RNA determine detection limits	32, 46
Quantification of gene expression	Analogue	Digital	4, 38
Analysis	Widely accessible with user-friendly software packages	More challenging; often requires a bioinformatician	34, 47, 48
Cost	Relatively cheap overall; predictable cost per sample	Generally more expensive; library prepn costs accumulate per sample; sequencing costs accumulate per lane (into which samples may be multiplexed)	49
Level of technical variability	Higher	Lower	40, 50
Sample size calculation method	Standard tools for calculation	Complex tradeoff between sample numbers, read depth, and cost	34
Presence of batch effects	Sometimes problematic between chips; very problematic between different arrays; well-developed software tools can compensate for batch effects to some extent	Mainly during library prepn; software tools to compensate for batch effect are evolving	51
Types of technical bias	Background and cross-hybridization bias; dye bias arising from samples labeled with different dyes	Length bias arising from differences in transcript lengths between genes and GC content bias, with uneven coverage in GC-poor or GC-rich regions	52–55

In theory, RNA-seq is much better suited for studies of host-pathogen interactions, although this is not always straightforward (31, 56). A major challenge is that the RNA from the pathogen may comprise only a tiny proportion of the total RNA isolated from a specimen, particularly in the case of bacterial infection. One solution is to use model systems, for example, genetically modified fluorescent pathogens, to allow cell sorting and selection of infected host cells (31, 32). An alternative is the specific enrichment of pathogen transcripts at the time of RNA extraction (32). Additional steps to maximize the capture of pathogen RNA, for example, enhanced lysis to release both bacterial and host RNAs from cells, and rRNA depletion to maximize the sequencing of mRNA may be needed (57).

These approaches have led to the identification of host-pathogen interactions at a cellular scale, such as the regulation of invasion-associated effectors and virulence genes by bacterial noncoding RNAs (31), but there is an increasing desire to apply dual RNA-seq to infections *in vivo*, and deep RNA sequencing is already achieving some success (58, 59). This has led to evidence that pathogen gene expression can vary within the host (59) and can be driven by the host response (58), providing a further impetus for this approach. Dual RNA-seq has also been applied to viral infections, allowing virus detection to be coupled to host transcriptome analysis (60), quantification of viral loads (61), and detection of variation in viral gene expression levels (62).

Eukaryotic pathogens generally contain more RNA and may have a greater capacity to change their gene expression in response to their host. Feasibility has been demonstrated, for example, in the gut of mice infected with whipworm (63) and the blood of P. falciparum-infected malaria patients (where about 10% of whole-blood reads mapped to the parasite) (25). In contrast, dual RNA-seq of brain tissue from mice infected with a different apicomplexan parasite, Toxoplasma gondii, showed a much lower proportion of reads (around 0.1%) mapping to the parasite genome (64), which likely reflects the very low abundance of parasite relative to host cells. Similarly, systemic infection with Candida albicans yielded so little fungal RNA from mouse kidneys that specific enrichment was necessary for pathogen RNA-seq (65). Therefore, obtaining appropriate data for simultaneous host and pathogen transcriptomic studies is highly dependent on the pathogen, the RNA content per pathogen, the sample type, and the pathogen load.

## TRANSCRIPTOMIC STUDIES OF MALARIA

### What Transcriptomic Studies Have Been Done on Malaria?

There are many transcriptomic studies of Plasmodium species *in vitro* and *in vivo*. Here we focus primarily on those using samples from humans with P. falciparum malaria and animal models of human infection, and we draw upon pivotal *in vitro* studies, where necessary, to give context. Recent reviews have addressed the application of transcriptomics to specific aspects of malaria immunology, vaccinology, and host-parasite interactions (66–68). We aim to provide a broader overview, integrating findings across disciplines and across host and parasite species and highlighting the potential of the simultaneous analysis of host and parasite.

**FIG 3:**
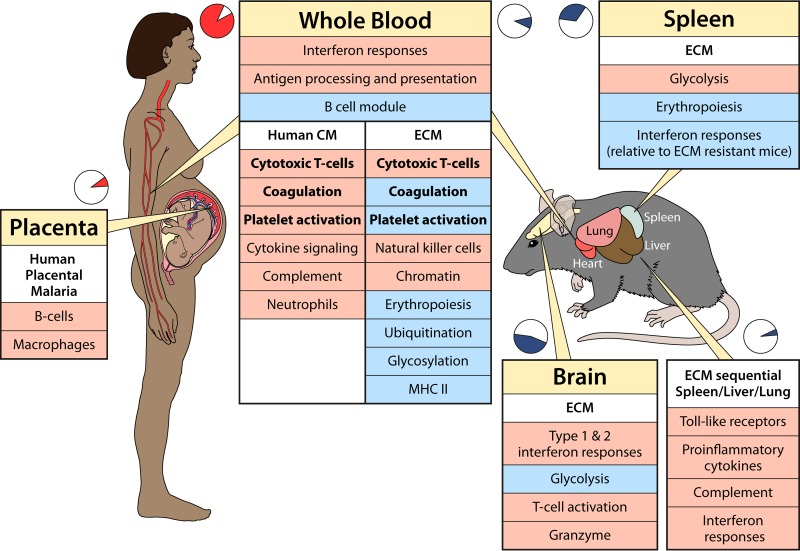
Transcriptomic host response to malaria in humans and mice. Shown is a comparison of selected features of the transcriptional host response to malaria in humans (left) and mice (right). Pie charts indicate how frequently each tissue has been studied for each species. Broad functional groups of genes are presented as upregulated (light red) or downregulated (light blue) in each species for uncomplicated disease and for cerebral malaria (CM) or experimental cerebral malaria (ECM). Common functional groups between humans and mice are marked in boldface type.

Over the 15 years since the earliest of these studies was reported, the available technologies have evolved considerably (Fig. 2). Most studies considered in this review used microarray technology, although there has been a recent proliferation of RNA-seq studies. Only two reported studies conducted dual-transcriptome analyses (25, 69). Here we synthesize the most important findings from these studies and consider their implications under five broad categories: technological advancement, basic biology, immune response, pathogenesis, and biomarkers. This synthesis is limited by the heterogeneity of experimental designs, technical and analytical approaches, and organs and species studied (Fig. 3), but despite this, some consistent findings and some clear holes in current knowledge emerge.

### What Have Transcriptomic Studies Taught Us about Malaria?

#### Technical challenges and solutions.

The first complete genome sequence of P. falciparum was reported in 2002 (70) and was soon followed by transcriptomic studies of parasite gene expression *in vitro* (71, 72) and *in vivo* (23). Draft genomes of rodent malaria parasites began to be reported at the same time (73, 74), and together, these resources opened the way for exciting analyses of host-parasite interactions in humans and popular experimental models. Since most transcriptomic studies have the implicit assumption that transcription and translation are tightly linked, ribosomal sequencing has only recently confirmed that this is indeed largely true for P. falciparum albeit with some evidence of additional translational regulation (75).

There are specific technical challenges associated with transcriptomic analyses of Plasmodium species, including intrinsic properties of the parasite genomes; the mixing of parasite and host RNAs, which accompanies parasitism; the variation in gene expression with progression though the developmental cycle; and the inaccessibility of parasites at certain stages of the life cycle.

The genome of P. falciparum has one of the most AT-rich compositions among all eukaryotes (70), which has made accurate sequencing, annotation, and assembly of the genome and transcriptome more difficult. High AT content creates PCR amplification bias and homopolymer tracks, which can bias quantitative analyses and makes read mapping more difficult because it results in more repeats. Plasmodium genomes also contain multigene families, which, particularly in P. falciparum, display extreme levels of genetic variation and present challenges for probe design and sequence mapping or assembly (19, 76). The classification of genes within one of these multigene families using domains that can be defined by PCR has enabled specific groups of the *var* genes of P. falciparum to be associated with clinical phenotypes (77). Other multigene families, such as *rif* and *stevor* (19), have not yet been characterized in such detail, and there is also genetic diversity in many other loci (78). Workaround solutions have been developed, such as custom arrays with probes designed to capture transcripts from multiple different parasite strains (79), but it is difficult to know how much diversity they really capture. Highly polymorphic genes present a particular challenge for reference-based RNA-seq analyses, and even though *de novo* assembly of transcripts is possible, the best way to accurately assemble, quantify, and compare expression levels between specimens is still uncertain.

Much of the Plasmodium life cycle is spent within host cells, and so another technical challenge is separating the mixed host and parasite RNAs that occur in biological samples. One of the first solutions was the simultaneous capture of host and parasite transcriptomes by using custom microarray designs with probes specific for each species (69). Next-generation sequencing (NGS) technology allowed alternative approaches to be applied, with the potential to separate signals from host and parasite RNAs (either physically or computationally) and then analyze them individually or in comparison with each other. The application of custom-designed nonrandom primers enabled the specific analysis of parasite transcripts by excluding human RNA, rRNA, and globin mRNA at the library preparation stage (80). However, unambiguous mapping of even relatively short reads to one species or the other has since been demonstrated to be possible when whole-blood host and parasite mRNAs were captured by using poly(A) selection and simultaneous sequencing (25).

*In vitro* parasite development can be synchronized through various treatments (81), but *in vivo*, parasites may coexist at different developmental stages. Plasmodium exhibits stage-dependent gene expression (discussed in more detail below), which can confound the interpretation of the transcriptome obtained from analysis of mixed stages. Several analytical approaches have been developed to try to estimate the developmental stage of parasites, including a maximum likelihood approach using global gene expression (82) and a more simplistic approach using single, stage-specific marker genes (83). Despite the clear rationale for these methods to be applied, it is notable that few transcriptomic studies of malaria have used them.

Parasite sequestration in the microvasculature is a pathognomonic feature of P. falciparum malaria (17, 18), and the extent of sequestration is one factor that contributes to differences in developmental-stage mixtures (84). Sequestration is strongly associated with pathogenesis, and so transcriptomic analyses of sequestered parasites may reveal mechanisms of severe disease. However, sequestered parasites are absent from the circulating blood and therefore are difficult to access. One approach has been to use formalin-fixed paraffin-embedded postmortem tissue blocks from patients who died of malaria, and these specimens enable sequestered parasite gene expression profiles to be obtained from brain and other tissues (85). This potentially opens the way for exciting dual RNA-seq studies on similar specimens (Fig. 2), which may reveal much more about the interaction of sequestered parasites with the host vascular endothelium and the tissues in which they are located.

#### Basic biology: parasite biology.

One of the most fundamental applications of transcriptomics to parasite biology has been the use of RNA-seq to improve the annotation of parasite reference genomes by identifying novel transcripts, verifying or correcting gene models, and identifying splicing sites (86, 87). This has proven particularly useful for the precise annotation of the P. falciparum genome and also produced major improvements in previously fragmented genomes of the common model rodent malaria parasites (88). Such a comprehensive annotation is a prerequisite for any attempt to relate quantitative gene expression to parasite biology.

Microarray and RNA-seq studies have also been fundamental for understanding the variation in gene expression that accompanies the complex life cycle of Plasmodium parasites and for identifying transcription factors that control its progression (71, 86, 89, 90). Throughout the intraerythrocytic cycle, there is a striking phasic variation in the expression of the majority of parasite genes (71, 86, 91), which rations protein production to occur only when the proteins are required. For example, genes involved in erythrocyte invasion are expressed only in mature schizonts so that when daughter merozoites are released into the blood, they are fully equipped to invade new red cells (71). Despite a conserved overall pattern of phasic variation, the expression of individual homologue genes is not so highly conserved among different Plasmodium species and shows the greatest variation at the stage of the greatest interaction with the host cell, during early-ring-stage development (91). The development of the gametocyte forms, required for the transmission of Plasmodium from vertebrates to mosquitoes, is accompanied by another unique gene expression profile and controlled by a master regulator transcription factor, AP2G (89, 90).

Since gene expression is so tightly linked to the developmental stage, it is not surprising that comparisons of gene expression levels between samples with asynchronous parasite populations (as often seen *in vivo*) can be misleading if no consideration is given to the composition of the mixture of parasite stages (82). However, assessment of parasite gene expression *in vivo* is very important, as there may be transcriptomic variation that is not seen under the standardized conditions used for parasite propagation *in vitro*. One of the first studies to attempt global gene expression analysis *in vivo* identified several different patterns in parasites drawn from malaria patients (92), and by analogy to the better-understood biology of Saccharomyces cerevisiae, these patterns were related to distinct physiological states (92). These states were also related to host factors, including cytokine profiles, which raised the intriguing possibility that they may represent a parasite response to the host environment. However, a subsequent reanalysis of these data suggested that much of the variation was due to differences in the mixtures of developmental stages between subjects rather than large changes in the gene expression of parasites at the same developmental stages (82).

Recently developed single-cell RNA-seq approaches offer the potential to overcome some of the problems with bulk analyses of mixed parasite populations (90), although the practical and technical challenges for achieving unbiased transcriptome analysis are significant. Most current methodologies are restricted to polyadenylated transcripts, losing information on small noncoding RNAs and some long noncoding RNAs (93). Transcript recovery rates can be low, even for deep sequencing (94), and the low RNA content in small microbes can further diminish recovery (95). The decision of whether to examine large numbers of cells at a low sequence depth or small numbers of cells at an increased depth depends very much on the motivation for the experiment (93, 96), yet carefully designed experiments with these limitations in mind are still revealing. For example, preliminary results suggest that even in synchronized parasite cultures, subtle variation in the developmental stage may create an illusion of sinusoidal patterns of gene expression during the erythrocytic developmental cycle of P. falciparum, when in reality, the pattern is much more discontinuous (97).

Descriptive analyses of the distribution of parasite developmental stages *in vivo* have provided useful information in their own right, allowing the timing of parasite sequestration to be pinpointed to around 22 h after erythrocyte invasion and providing insights into gametocyte development (98). Very-early-ring-stage gametocytes and mature gametocytes were detectable in blood, while the intermediate stages of gametocyte development were not detectable, consistent with the concept that this occurs in sites of sequestration (20).

Comparisons between Plasmodium species can give valuable insight into parasite biology. All Plasmodium genomes sequenced to date have multigene families in the subtelomeric regions of most of their chromosomes (70, 88, 99–104), which encode proteins expressed close to or on the surface of iRBCs. Depending on the species, up to 30% of the parasite genome is dedicated to these multigene families (105), suggesting that they have important roles (99). One of the best-known multigene families is the *var* family of P. falciparum, which encodes around 60 different copies per parasite of P. falciparum erythrocyte membrane protein 1 (PfEMP1) variants, antigenically diverse proteins expressed on the surface of iRBCs (19). The transcriptional control of *var* expression results in antigenic variation and immune evasion (19, 106–109). *var* genes are also involved in the interaction with and adhesion to host cell surfaces, such as adhesion to the vascular endothelium (resulting in sequestration) or binding to uninfected RBCs (a phenomenon known as rosetting), both of which are correlated with virulence (19, 108). While the *var* gene family is unique to P. falciparum, the *PIR* (Plasmodium interspersed repeat) multigene family can be found in every Plasmodium genome that has been sequenced so far. This vast gene family includes large numbers of gene loci in each species, including *stevor* (∼40 loci) and *rif* (∼180 loci) in P. falciparum, ∼180 loci in P. berghei, ∼800 loci in P. yoelii, ∼200 loci in P. chabaudi, ∼68 loci in P. knowlesi, ∼1,200 loci in P. cynomolgi and P. vivax, ∼250 loci in P. malariae, and nearly 2,000 loci in P. ovale (99, 100, 105, 110, 111).

Transcriptomic studies have revealed fascinating insights into the role of this gene family in P. chabaudi infection. The repertoire of expression of the *P. chabaudi PIR* (*Pc-PIR*) genes is strongly influenced by whether mice are infected by a mosquito bite or serial blood passage, and these modes of transmission also determine parasite virulence (112). Serial blood passage leads to the expression of a single dominant *Pc-PIR* gene in blood-stage parasites and increased virulence, while mosquito transmission seemingly reverses the constraints imposed by serial blood passage, allowing a greater repertoire of *Pc-PIR* genes to be expressed, and parasites are less virulent. The mechanism by which this profile is reset is unknown but is hypothesized to have an epigenetic basis (112, 113). An interesting parallel is that the diversity of the array of *var* genes expressed in an infected human host may also decrease as virulence increases following serial blood passage (112, 113).

The *Pc-PIR* genes also play a role in the establishment of chronic or persistent infection, which is important for parasite transmission. Comparisons of parasite transcriptomes in acute and chronic P. chabaudi infections revealed the differential expression of about half of the *Pc-PIR* genes. Most of these genes were upregulated during the acute phase of infection (113). Interestingly, this differential expression was not a consequence of immune selection but rather reflected the expression of specific clusters of *Pc-PIR* genes that were consistently associated with either acute or chronic infection, suggesting programmed rather than selected expression and another unknown mechanism, which will be important to delineate (113, 114).

These findings once again raise the question of whether the control of parasite gene expression *in vivo* is well represented in laboratory-adapted parasites grown for many generations *in vitro*, which are the basis for much of our knowledge about parasite biology. Existing evidence (albeit from small studies) suggests that parasite gene expression is generally well conserved between laboratory-adapted P. falciparum strains and parasites directly sampled from naturally infected humans (115). However, the expression of genes encoding molecules exported to the red cell surface, including those of the *rif* and *stevor* families, appears upregulated *in vivo* compared to that in laboratory strains. Among subjects with severe malaria, greater departures from the *in vitro* transcriptome have been described (116), perhaps suggesting that the more perturbed the host environment, the more the parasite must adapt its gene expression. Adaptation of parasite gene expression to the host environment was recently demonstrated when infected mice were fed either low-energy or normal diets, with changes in the parasite transcriptome leading to the identification of the putative serine/threonine kinase KIN as a parasite sensor of host nutritional status and a regulator of parasite growth (117).

Controlled approaches comparing synchronous parasites from recently culture-adapted field isolates and long-term-laboratory-adapted strains have also confirmed variation in the expression of genes encoding parasite proteins exported to the red cell and to its surface as well as higher expression levels of genes coding for sexual-stage proteins in field isolates. In a study of Kenyan parasite isolates, this differential expression appeared to be partly attributable to genetic changes, particularly copy number variation (118). Interestingly, studies of inbred mice infected with the nonlethal parasite P. yoelii 17X showed a considerable conservation of parasite gene expression over time and between hosts, even among hosts with different immune statuses. The greatest variation occurred at peak parasitemia, when there was maximal reticulocytosis, and the parasite may require different proteins for the most efficient growth in these young RBCs (119). Taken together, these findings suggest that many variations in parasite gene expression *in vivo* may be determined by the necessity for an optimal interaction with host erythrocytes. The question of whether additional large-scale variation in gene expression plays a causal role in severe disease in humans remains to be fully resolved.

#### Immune response.

##### (i) Naturally occurring immune responses.

The complex life cycle of Plasmodium means that the immune response to malaria needs to be understood as a series of responses to spatially, temporally, and antigenically distinct life cycle stages (14) (Fig. 1). Superimposed on this, the intensity, duration, and timing of previous infection can modulate acquired immunity (15, 21). Enormous antigenic variation creates considerable challenges for the immune system. Although a huge amount has been learned about immune responses to malaria in both humans and animal models (120), most of our understanding comes from reductionist studies, and the integrative understanding of the immune responses at a systems level remains rudimentary.

Transcriptomic studies therefore play an important role in and are potential building blocks for an integrated description of immune responses to malaria. It would be ideal to obtain serial samples from all relevant tissues, starting before inoculation by a mosquito bite, through the presymptomatic and symptomatic phases of infection, and onwards until the time of death, resolution, or persistent infection. This may be possible in animal models (although it has not yet been done), but human studies are often constrained by the limited availability of any sample type other than blood and the practical and ethical challenges of longitudinal sampling (121). Thus, we must piece together a likely sequence of events from limited samples from humans and data from experimental malaria infections in other species.

Insights into some of the earliest immune responses to blood-stage parasites in humans come from controlled-infection studies, whereby malaria-naive individuals are infected by a mosquito bite or the inoculation of blood-stage parasites and then intensively monitored for the detection of parasites in their blood (122, 123). An early microarray study showed that large changes in peripheral blood mononuclear cell (PBMC) gene expression were already apparent at the time of the first detection of parasites on a blood film, which for most subjects preceded the onset of symptoms (124). These changes included the upregulation of cell surface and intracellular pattern recognition receptors, proinflammatory cytokines, phagocytic and scavenger receptors, and NADPH oxidase components, together indicating a coordinated activation of multiple components of the innate immune response. Interferon gamma (IFN-γ) signaling pathway, interleukin-1β (IL-1β) signaling, and glycolysis pathway genes were prominently activated early in infection, as were genes involved in antigen processing and presentation for major histocompatibility complex class I (MHC-I) and MHC-II; however, the upregulation of the expression of the IL-1β receptor and heat shock protein genes was limited to subjects who developed fever (124). Looking slightly later in infection, when previously malaria-naive subjects first developed fever, broadly similar findings were observed by using RNA-seq, although the downregulation of T- and B-lymphocyte genes was noted (125).

Gene expression profiles from naturally infected individuals at the time of clinical presentation with acute uncomplicated malaria (UM) show many similarities with those of presymptomatic experimentally induced infections (24, 25, 124–126). However, the additional induction of genes related to interleukin-10, mitogen-activated protein kinase activation, and Fas ligand-induced apoptosis was detectable in PBMCs of naturally infected, symptomatic Cameroonian adults (124). Type I interferon-related genes were highly induced by infection in comparison to healthy controls and in comparisons of paired acute- and convalescent-phase samples from Brazilian patients (127, 128). Comparison of whole-blood gene expression from symptomatic previously naive individuals with that from symptomatic Malian adults showed less upregulation of interferon responses but greater upregulation of B-cell receptor signaling in malaria-experienced individuals (125). Prominent neutrophil-associated signatures were additionally found in whole blood from symptomatic children (24, 126). The most perplexing differences observed between studies of symptomatic individuals relate to opposing changes in MHC-, T-cell-, and B-cell-associated genes (24, 124–126). These conflicting findings may represent genetic or environmental differences between the different comparison groups, differences in parasite loads between subjects (126), or changes in the proportions of leukocyte subpopulations in infection, the effect of which is dependent on whether RNA is extracted from PBMCs or whole blood.

Differences between the transcriptional profiles seen in cases of uncomplicated and severe malaria have been less studied. A paired comparison of 5 individuals who first presented with severe malaria and later returned with an episode of uncomplicated malaria found that IFN pathway and T-cell response genes were more highly expressed in the uncomplicated episodes than in the severe episodes (129). Although that study did not specifically consider the association of gene expression with parasite load, other observations (126) suggest that this difference may be largely a consequence of the lower parasite load at presentation with uncomplicated malaria.

Since natural exposure often involves repeated Plasmodium infections, transcriptomic approaches have been used to understand the consequences that one or more episodes of malaria may have on subsequent responses to Plasmodium or other pathogens. Comparison of PBMC transcriptomes of Malian children 7 days after treatment for the first acute episode of the malaria season with transcriptomes just before the onset of the malaria season revealed the downregulation of inflammatory genes but the upregulation of genes expected to facilitate microbial killing and the activation of adaptive immunity (130). Stimulation of these PBMCs with infected RBCs also resulted in lower expression levels of inflammatory genes but higher expression levels of microbial killing and adaptive immunity genes in the samples from 7 days after infection. Those findings suggest that one episode of infection might be able to program a more advantageous response to subsequent infection. However, there is also abundant evidence that acquisition of immunity is inefficient, and repeated malaria exposure impairs heterologous immune responses and increases susceptibility to other infections (131, 132). Dysfunctional atypical memory B-cell populations have been described for other infections associated with poor antibody production (133), and transcriptome analysis has been central to defining atypical memory B-cell populations related to chronic malaria infections, which are defective in immunoglobulin production and denoted by the surface expression of FCRL5 (134, 135).

There are few data on the evolution of changes in the blood transcriptome with the progression of infection in animal malaria models, although data available for P. chabaudi infections suggest that there are considerable overlaps with the human blood transcriptome in uncomplicated pediatric malaria (126, 136, 137) (Fig. 3). Pathway-level similarities include the upregulation of IFN response, antigen presentation, and proteasome-related genes; the downregulation of B-cell genes; and gene-level overlap of the upregulation of Fc receptors. However, T-cell signaling was upregulated in mouse whole blood, in contrast to the lower expression levels in human whole blood reported by those same authors (126).

In a different rodent infection, P. berghei ANKA, the immune responses in liver, spleen, lungs, and brain were monitored at sequential time points (69). Although that study primarily aimed to investigate the pathogenesis of experimental cerebral malaria (ECM), the sequential immune response over time is noteworthy because it varied by organ and by mouse species. Overall, there were many similarities to the human immune response genes induced by malaria, particularly in spleen, liver, and lung, where Toll-like receptor (TLRs), proinflammatory cytokine, interferon-inducible, and complement-related genes were induced. Unfortunately, that study did not include blood transcriptome data, which might have allowed a better comparison with human data and inference of changes in blood gene expression that may arise from the migration of cells to and from different organs.

The accessibility of fresh organs from mice permits more-refined transcriptomic analyses on specific cell populations isolated from these organs, avoiding confounding due to changes in cell populations within a whole organ or blood. Splenic cells have been the primary focus of such analyses, since the spleen is a major site of interaction between parasites and innate and adaptive immune cells, which control human and rodent malaria infections (138). For example, purified CD11c^+^ splenic dendritic cells (DCs) were examined at different time points during P. yoelii 17XNL-infected BALB/c mice to resolve a controversy surrounding their function in malaria (139). At the time of that study, opposing roles had been proposed, with DCs on the one hand enhancing innate immune responses and initiating adaptive immune responses (140) and on the other hand mediating the immunosuppressive effects of the parasite product hemozoin (141). Transcriptomic analyses revealed several distinct patterns of gene expression over time, with many immune-related genes showing sustained levels of transcription at early and late time points during infection but a notable smaller group showing differential regulation between time points (139). Perhaps important to understanding the controversy around DC function, expression levels of *il-10* were higher and expression levels of *il-6*, *il-12*, and *ifng* were lower later during infection. Genes involved in the cell cycle, glycolysis, and purine metabolism were also extensively modulated by P. yoelii infection, in contrast to previously described “common” DC maturation signatures, suggesting that DC behavior in malaria may not easily be inferred from that observed in other situations (139).

Analyses of purified splenic CD4 T cells have also contributed to understanding their role in malaria, this time in the lethal P. berghei ANKA model (142). In this model, CD4 T cells enhance pathogenicity and are ineffective at providing T-cell help to constrain the parasite load. Their early transcriptional response to infection was dominated by interferon gamma and, unexpectedly, type I interferon response genes, which led to functional studies demonstrating that type I interferon was responsible for suppressed CD4 T-cell function (142). An even deeper understanding of splenic CD4 T-cell biology followed, using single-cell isolation and RNA-seq combined with fate mapping to determine how CD4 T-cell clones differentiate into either T helper 1 or T follicular helper cells (143). Cell cycle and glycolysis genes, a recurring feature in analyses of splenic tissue (139, 144, 145), were upregulated in these cells around the time of fate determination at day 4 of P. chabaudi infection, and subsequent fate was determined by interactions with B cells or monocytes, which favored T follicular helper or T helper 1 development, respectively (143). Undoubtedly, cell fate mapping and single-cell sequencing will add further essential detail and complexity to our understanding of malaria immunology in the future.

##### (ii) Vaccine-induced immune responses.

Transcriptomic approaches have yielded particular success in identifying vaccine-induced protective immune responses for viral infections such as yellow fever and influenza (146, 147), catalyzing the development of the new discipline of systems vaccinology (148, 149). However, the development of a protective malaria vaccine has been an arduous process because a vaccine has to perform considerably better than naturally induced immunity, and until a partially protective vaccine became available, it was not possible to begin identifying correlates of vaccine protection (14). Various strategies, ranging from intravenous whole attenuated sporozoites to recombinant proteins and DNA-based vaccines, have been evaluated in attempts to target single or multiple parasite life cycle stages (reviewed in references 14, 150, and 151).

To date, the only vaccine to have achieved licensure and enter into pilot implementation in countries where malaria is endemic is RTS,S/AS01 (150, 152). This vaccine has demonstrated only modest efficacy and durability in clinical trials in African children, but it has the potential to make a substantial public health impact (153–156). RTS,S/AS01 is a preerythrocytic vaccine, so when effective, it either prevents or delays the development of blood-stage parasites and clinical disease (14, 150). The imperfect protection afforded by RTS,S has allowed transcriptomic studies of unprotected and protected individuals to be undertaken to characterize vaccine responses, which predict subsequent protection against experimental challenge. In peripheral blood mononuclear cells, plasmablast-associated transcriptional signatures, cell cycle genes, and type I interferon genes correlated positively with immunogenicity (antibody titers to circumsporozoite protein [CSP]) and vaccine-induced protection, while natural killer (NK) cell genes were negatively correlated with both outcomes (157, 158). Looking at the role of the genes by functional enrichment, immunoproteasome, cell cycle, and apoptosis functions were associated with protection (158), and more-focused analyses have highlighted that vaccine-induced interferon signaling may also predict protection (159). Decreases in this response signature shortly after the final vaccination were associated with a lack of protection (160).

While RTS,S is the most advanced vaccine, it is not the only preerythrocytic vaccine to be developed, and transcriptomic correlates of protection for RTS,S might be compared with those induced by other vaccines in order to find universal correlates of protection. In a schedule of priming with an adenovirus-vectored vaccine and boosting with two doses of RTS,S/AS01, a strategy designed to elicit better CD8 T-cell responses, transcriptional correlates of protection were not identical to those of RTS,S/AS01 alone (157). In the prime-boost approach, innate responses to the vaccine appeared more important, with TLR signaling, dendritic cell, and antigen presentation gene expressions all correlating with protection. The most consistent common feature between this regimen and RTS,S/AS01 alone was a negative association between protection and NK cell gene expression (157). However, these vaccine regimens also produced different immunological correlates of protection, with polyfunctional CD4 T cells rather than anti-CSP antibodies emerging as being the most important for the prime-boost regimen despite similar protective efficacies. *Ex vivo* restimulation of samples from two other studies with the vaccine antigen, using either two doses of RTS,S followed by modified vaccinia virus Ankara (MVA)-vectored CSP or two doses of DNA multiple-epitope thrombospondin-related adhesive protein (ME-TRAP) followed by MVA-vectored ME-TRAP, showed that the small number of protected subjects were characterized by the upregulation of interferon-induced and antigen presentation genes and the downregulation of hematopoietic stem cell and myeloid cell genes (161). Despite some common themes in protection-associated gene signatures from those studies, the ideal response to provide sterile protection against preerythrocytic parasite stages in humans remains to be characterized.

##### (iii) Gene expression profiles associated with asymptomatic infection.

In countries where malaria is endemic, it is common for individuals to have asymptomatic parasitemia (11, 12). The likelihood of an infection being asymptomatic increases with age (12) and decreases with parasite load (27), but many believe that this must also involve the active regulation of the immune response, since parasite loads tolerated under high-transmission intensity are much higher than those causing fever under low-transmission intensity (162). It is therefore intriguing that no significant transcriptomic response to asymptomatic infection was detected by RNA-seq despite comparing paired samples prior to infection with those during infection in Malian adults albeit with only 5 subjects per group (125).

It is tempting to speculate that this may indicate that none of these individuals had reached sufficient parasitemia to trigger a response and that the set points for such a response may differ between malaria-experienced and previously naive individuals. This concept may be supported by data from a recent study comparing the transcriptional responses of asymptomatic adolescent men from two sympatric ethnic groups in Burkina Faso with similar peripheral blood parasite densities (163). Comparison of purified monocyte transcriptomes between 7 uninfected and 2 infected individuals of the Fulani tribe showed dramatic differences in gene expression, whereas the same comparison for individuals of the Mossi tribe showed negligible differences. Fulani are well known to have relative protection from malaria (164), and the authors of that study speculated that these results might indicate an immune response that is more poised for activity upon infection in the Fulani than in the Mossi. Further work in larger studies restricted to individuals with persistent asymptomatic infection will be important to investigate this issue further.

Taking a different approach, a longitudinal study identified a Vδ2^+^ subset of γδ T cells as being reduced following chronic malaria exposure, and their gene expression was investigated (165). Interestingly, their basal expression levels of numerous immunoregulatory genes were found to be increased, and their transcriptional inflammatory response to infected RBC stimulation was diminished, concordant with their association with a diminished likelihood of symptoms upon infection. This suggests that the infection-induced attenuation of this cell population may help to explain why repeatedly exposed individuals become less likely to develop symptoms.

##### (iv) Effect of host immunity on parasite gene expression.

To date, relatively little is known about how preexisting host immunity (naturally acquired or vaccine induced) alters parasite behavior. Transcriptomic analyses, particularly dual RNA-seq of host and parasite, could provide insights into this question. However, some surprising findings have arisen from studying the more fundamental question of whether the presence or absence of specific components of host immunity alters parasite gene expression. A common assumption has been that the expression of different members of parasite variable gene families (such as *PIR* genes) enables immune evasion and is at least partly determined by immune selection. For the *Pc-PIR* genes of P. chabaudi, this appears not to be the case. Transcriptomic comparisons of parasite gene expression levels in mice with acute and chronic infections demonstrated that the establishment of chronic infection was indeed associated with a clear shift in *Pc-PIR* gene expression, but this was not influenced by the removal of immune selection in mice without T cells (TCRα^−/−^ mice) or without B cells and antibodies (μMT mice) (113). This finding conflicts somewhat with human data that suggest that *var* gene expression is affected by preexisting humoral immunity (166).

#### Pathogenesis.

Malaria typically causes fevers, headache, myalgia, rigors, cough, and abdominal pain, features which are similar to those of many other systemic infections (11, 12). Laboratory tests often show anemia, thrombocytopenia, and increased levels of acute-phase response proteins (12, 18). Features associated with an increased risk of death include coma, renal failure, metabolic acidosis, hypoglycemia, respiratory distress, and severe anemia (167, 168). The pathogenesis of the clinical and laboratory features of malaria is incompletely understood, and this is especially so for the progression from uncomplicated to severe malaria (13). Although transcriptomic approaches have the ability to broaden our understanding of the changes that occur in the host and the parasite during infection, relatively few studies have sought to associate gene expression with specific features of human disease (24, 25, 124, 169–171). In contrast, transcriptomic studies with animal models have frequently been used to try to provide an understanding of the pathogenesis of severe malaria. However, the interpretation of the results generated in these models is dependent on understanding both the relevance of the model to human disease and the experimental design, most importantly the severe and nonsevere comparison groups.

The most common models use inbred strains of mice of specified ages and sexes. Unlike natural malaria infections in humans, the outcome of these models tends to be extremely consistent for any given combination of parasite and mouse strain. For example, the P. berghei ANKA strain causes a neurological syndrome described as ECM (172) in C57BL/6 and CBA mice but does not cause ECM in BALB/c mice (173). The closely related strains P. berghei K173 and P. berghei NK65 do not usually cause ECM in any of these mouse strains (172). Investigators have generally taken one or more approaches to identify gene expression associated with severe disease: (i) comparison of susceptible and resistant mouse strains infected with the same parasite strain (69, 173–177); (ii) comparison of the same mouse strain infected with different parasite strains (137, 178); (iii) time course analyses to identify differences in gene expression occurring before and after the onset of severe disease (69, 126, 136, 144, 175, 177, 179); and (iv) comparison of rare, less severely affected mice with their more severely affected counterparts of the same strain infected with the same parasite for the same duration (136, 176). All of these approaches potentially have limitations because it is difficult to disentangle expression differences associated with the genetic background from those causing severe malaria. Furthermore, features that are critical for valid comparisons in models with different mouse or parasite strains, such as temporal changes in parasitemia or total body parasite load in individual mice, have been inconsistently reported. Despite these limitations, common features emerge from transcriptomic studies using rodent models regardless of the experimental approach (Fig. 3).

Unfortunately, studies of pathogenesis in humans and animal models have often been conducted in relative isolation, and the relevance of animal models, such as ECM, to human disease is often debated because of differences in key histopathological features, such as parasite sequestration (172, 180). Transcriptomic studies have the potential to allow global comparisons of host and parasite gene expression between these models and human specimens, but this has not yet been done in any formal way. There are no studies to date comparing human gene expression from organs such as brain or lung in severe and uncomplicated malaria cases (Fig. 3). In contrast, relatively few animal studies have investigated gene expression in blood (126, 136, 137, 173), and the majority of those studies focused on brain and spleen gene expression. Thus, synthesis of findings in rodent models with those in humans is challenging (Fig. 3).

##### (i) Cerebral malaria and experimental cerebral malaria.

The pathogenesis of human cerebral malaria (CM) has been extensively debated because it is currently impossible to prove the dependency of the syndrome on any specific pathogenic mechanism. In contrast to the widely used P. berghei ANKA C57BL/6 ECM model, human CM may not even be a single entity but may have several pathological subtypes (181–184). The first of these subtypes is not CM at all but “false CM,” with coma being due to another cause (infectious or noninfectious) in the presence of incidental parasitemia (181). In resource-poor settings, it is difficult to exclude all other possible causes of coma, and so they may be misclassified as CM. A relatively common and specific feature of CM that is not present in false CM is malarial retinopathy, which colocalizes with the sequestration of parasites in the retinal blood vessels (181, 185). This has been used to define children with true CM, but it has become apparent that coma in some children without retinopathy is also at least partly caused by malaria (182, 183, 186).

###### (a) Host response.

Few studies have compared the human or mouse blood transcriptomes between CM and uncomplicated malaria (129, 170, 171) or between severe malaria phenotypes (187). In Malawian children initially treated for CM and subsequently reattending with an uncomplicated malaria episode, paired analyses of whole blood showed a striking upregulation of type I interferon-associated gene expression at presentation with uncomplicated malaria compared to the episode of severe malaria (129). Differential type I interferon responses were also a feature detected in a larger study comparing Malawian children with retinopathy-positive and -negative CM, along with a significant enrichment of cell adhesion and extracellular matrix pathways and numerous neutrophil-related transcripts being upregulated in the retinopathy-positive subjects (187). There was also an enrichment of pathways related to coagulation, platelet activation, and cytokine signaling (187), consistent with the well-described coagulopathy and inflammation that accompany CM (188, 189). Supporting the relevance of the transcriptomic findings, concentrations of neutrophil primary granule proteins (elastase and myeloperoxidase), tumor necrosis factor, monocyte chemotactic protein 1, and interleukin-10 were higher in plasma of retinopathy-positive subjects, whereas the concentration of IFN-α2 (a type I interferon) in plasma was higher in individuals with retinopathy-negative CM (187).

Using more-relaxed definitions of cerebral symptoms, a study in Mali that included 5 children with prostration or coma found higher expression levels of complement, Toll-like receptor, and cytotoxic-T-cell genes than those in 5 children with uncomplicated malaria (171). Many of these findings parallel those for the blood transcriptome in ECM, obtained from comparisons of susceptible and resistant mouse strains infected with P. berghei ANKA (173) (Fig. 3). Common differentially expressed genes in ECM include the downregulation of those associated with erythropoiesis, cell surface glycosylation, ubiquitination, MHC-II, platelet-related, clotting-related, and plasmacytoid dendritic cell-related genes (many involved in type I interferon signaling) in ECM (173). In contrast, there was an upregulation of natural killer cell- and cytotoxic-T-cell-related genes, the latter of which is consistent with the known dependency of ECM on CD8 T cells (173). However, changes in neutrophil gene expression signatures were not seen in ECM (173). A different perspective comes from a recent analysis of human PBMCs rather than whole blood, where comparison of genes associated with other neurodegenerative diseases between 7 children with CM and 8 with uncomplicated malaria suggested that protein aggregation pathways may be activated and important in CM (170).

In contrast to the limited studies on blood, multiple transcriptomic analyses of mouse brain tissue have been conducted and have yielded a fairly consistent picture of gene expression associated with ECM (69, 144, 174, 175, 177–179) despite the variety of experimental approaches discussed above. Although the brain parenchyma makes up the majority of specimens for gene expression analysis, it is composed of multiple cell populations, and additional cell types may actively or passively become enriched in the blood vessels and parenchyma during infection. Few studies specified whether brains were perfused prior to RNA extraction, but this method of flushing out nonadherent cells from the vasculature could result in substantial differences in gene expression due to the removal of intravascular leukocytes and immature RBCs. As evidence of this, several studies found transcriptional signatures of suppressed erythropoiesis (144, 176, 179), which likely reflects analyses of cells within the brain vasculature and mirrors findings in peripheral blood (173). The clearest consistent finding was the association of immune response and defense pathways with ECM (69, 144, 174–178). Specifically, genes associated with both type I and type II interferon signaling were enriched and upregulated in ECM versus comparators (69, 144, 174, 175, 178, 179). Genes associated with T-cell activation and granzyme were also enriched in several studies, consistent with their known role in ECM (144, 174–178, 190).

In some comparisons, an upregulation of type I interferon responses was found to precede the onset of cerebral pathology (69, 144), which is possibly related to sequential activation in different cell types, because isolated microglial cells showed prominent type I interferon gene expression profiles only after the onset of ECM (191). The prominence of type I interferon responses in those studies, associated with decreases in the levels type I interferon response genes in peripheral blood (173), leads us to suggest that there is likely a redistribution of cells producing type I interferon and/or a sequential pattern of upregulation followed by downregulation of type I interferon signaling, which varies in its timing by organ, progressing from peripheral blood to the brain vasculature to the brain parenchyma.

Beyond implicating immunopathological mechanisms initiating ECM, brain transcriptomes have also revealed possible explanations for neurological dysfunction. The increased expression and activation of apoptosis pathways were observed in several studies, along with a variety of cellular stress response pathways (175–178). However, pathological studies of ECM suggest that apoptosis is unlikely to be a major cause of ECM because it is an infrequent event, rare in parenchymal cells, and when occurring in vascular endothelial cells, it is not associated with adjacent edema or hemorrhage (180). One time course comparison of ECM-susceptible and -resistant mice reported evidence for an early downregulation of metabolic processes, such as glycolysis, in the brains of susceptible mice, which may plausibly contribute to reversible neurological dysfunction (177). However, many of the changes in gene expression associated with ECM may represent upstream events or noncausal associations, and it is possible that transcriptional changes actually play little role in the final neurological manifestations. A comparison of brains of wild-type C57BL/6 mice with ECM to those of resistant CD8 T-cell-deficient and perforin-deficient mice revealed smaller sets of differentially expressed genes than in a comparison with resistant BALB/c mice (176). In fact, it was striking that only 9 genes differed between perforin-deficient mice and wild-type mice with ECM, yet the perforin-deficient mice did not develop ECM, suggesting that the expression levels of very few genes need to change to produce the final neurological syndrome (176).

The spleen plays important roles in the innate clearance of parasites, the adaptive immune response, and erythropoiesis (in mice) (22, 138). Splenic gene expression has been investigated in both ECM and non-ECM severe rodent malaria models. Consistent with findings for other tissues, erythropoiesis genes were suppressed as ECM progressed (144) and were also suppressed in other lethal infections (discussed below). Metabolic pathway changes in the spleen accompanied the progression of infection, such as increased expression levels of glycolytic enzymes detected in whole spleen (144) and purified splenic CD11c^+^ dendritic cells (139), which may well represent metabolic switches necessary for immune cell proliferation and function. The induction of interferon-responsive genes in the spleen was found over the course of P. berghei ANKA infection in ECM-susceptible mice (144) but was greater at both baseline and late in infection in ECM-resistant mice, adding further complexity to understanding the roles of interferons in promoting or preventing ECM (69).

In an attempt to achieve a more integrated understanding, sequential changes in gene expression in spleen, brain, lung, and liver were examined in comparisons between ECM-susceptible and -resistant mice (69). Large groups of immune response genes showed consistent differences between mouse strains at all time points, but there were also clusters of immune response genes that became differentially expressed in different organs at different times, starting with the liver and later with the spleen and lungs. These temporally differing clusters may reflect the ability of the ECM-resistant BALB/c mice to mount earlier organ-specific responses to the parasites, but it remains unclear how this might prevent subsequent damaging responses in the brain (69).

###### (b) Parasite factors.

Attempts to link parasite gene expression to the pathogenesis of CM have supported a link between the expression of specific *var* genes and severity but beyond this have been rather inconclusive. Targeted analysis of the *var* transcriptome revealed that severe malaria (both CM and severe anemia) was associated with high expression levels of *var* genes encoding PfEMP1 variants with cysteine-rich interdomain regions predicted to bind to the endothelial protein C receptor (192), consistent with previous functional analyses highlighting the importance of this interaction in severe malaria (193, 194).

Analysis of P. falciparum isolates from 58 Malawian CM patients revealed considerable variation in parasite transcriptomes between subjects (116), with some showing marked departures from profiles observed *in vitro* (92). The strongest determinant of differences in these profiles was peripheral blood parasitemia (116). Subsequent combination of those data with additional gene expression data from subjects with uncomplicated malaria suggested that CM-associated parasites might show increased expression levels of genes that modify cytoadhesion and the rigidity of infected erythrocytes, exported proteins, and erythrocyte invasion proteins (98). Although highly plausible, that same study also highlighted the confounding effect of variation in the parasite developmental stage (98), and a smaller study that directly addressed this issue did not find any significant residual differences between groups with severe (including CM) and uncomplicated malaria (82).

Parasite gene expression in ECM has also been examined by using custom microarrays, but at that time, functional annotation of the P. berghei ANKA genome was rather limited (69). Nevertheless, organ-specific differences in parasite gene expression were detected, with the lung having the greatest detectable parasite gene expression, enriched in heat shock, ribosomal protein, and proteasome genes (69). Interpretation of such findings is challenging because differences may simply reflect differences in the distributions of parasite developmental stages associated with different organs, and a more refined analysis would be required to identify true differences in parasite gene expression in different tissues and to relate these differences to the already complex patterns of organ-specific host gene expression.

##### (ii) Other malaria phenotypes.

CM is the most studied severe malaria phenotype, but other life-threatening manifestations include severe anemia and respiratory distress (167). Respiratory distress is usually due to acidosis in children and reflects compensatory hyperventilation to raise the blood pH by the exhalation of more carbon dioxide (13). In adults, malaria-associated respiratory distress often represents true lung pathology with a picture similar to those of acute lung injury and acute respiratory distress syndrome (12). Severe anemia is probably the most common severe manifestation of malaria in very-high-transmission settings (13). If prompt blood transfusion and antimalarial treatment are available, the mortality rate can be low (195). Malaria in pregnancy is a special case that can result in severe disease manifestations in the mother but also placental dysfunction and adverse outcomes for the fetus ranging from abortion or stillbirth to growth retardation and premature birth (196). Non-ECM lethal animal models mostly lead to death through a combination of severe anemia and other organ dysfunction, which may include lung and liver pathology (197).

###### (a) Host response.

Fever is one of the key clinical features of malaria, but there is great variation in the temperatures at the time of clinical presentation among individuals infected with the same parasite species, likely reflecting the parasite load, synchronicity, and how recently iRBCs have ruptured and released parasite material (198). It is curious that body temperature has been the most common clinical variable analyzed for an association with global gene expression in humans, because it is likely to be confounded by many factors and is not useful for the prediction of clinical outcome. In whole blood, the expression of neutrophil-related (24) and lysosome-related (25) genes has been significantly associated with body temperature in acute malaria, while in PBMCs, heat shock proteins, interleukin-8 (a chemokine that promotes neutrophil chemotaxis), and interleukin-1β were significantly associated (124, 169). Unfortunately, no statistically significant associations of gene expression with the severity of anemia or with platelet counts have been identified in the few small studies examining these more important laboratory markers of pathogenesis (24, 169). The potential for the correlation of gene expression with clinical and laboratory features of malaria pathogenesis has not yet been fully exploited.

Pathogenicity-associated whole-blood host transcriptional profiles in mice have predominantly been assessed during P. chabaudi infections (136, 137). Comparisons between virulent CB strain and less virulent AS strain infections showed clusters of differentially expressed genes that had functional associations with platelet aggregation in dying mice and a more pronounced anemia signature and a neutrophil-dominated lung inflammation signature in CB-infected mice (137). Infection with the P. chabaudi AJ strain alone was analyzed in an innovative time course study (136) using gene expression to describe trajectories from health to illness and thence to either recovery or death. Intriguingly, in this model, the nadir of health in mice was preceded by NK cell gene expression but also showed transcriptional evidence of depressed erythropoiesis. A similar sequence of gene expression dynamics was inferred in humans by mapping sequential mouse data onto patterns of gene expression from a cross-sectional study of subjects with uncomplicated malaria (126). This novel approach may represent an important advance because it is almost impossible to examine sequential gene expression in humans with untreated symptomatic malaria, but consequently, robust validation is also challenging.

In other mouse models, analysis of the spleen transcriptome revealed reduced expression levels of genes associated with erythropoiesis at equivalent parasitemias in lethal P. berghei N67C infection compared with nonlethal P. berghei N67 infection (199) and the relative suppression of erythropoiesis for the severity of parasitemia in lethal P. yoelii 17XL infection versus nonlethal P. yoelii 17X infection (145). Type I interferons were particularly upregulated in spleen and shown to contribute to an enhanced control of parasitemia in nonlethal P. berghei N67 versus P. berghei N67C infection (199). However, this is not necessarily a universal mechanism for protection from severe disease. In a rat model using P. berghei ANKA (which does not cause ECM in this host), young rats failed to control parasitemia and died, while older rats controlled parasitemia, dependent on the differential expression of a small set of genes in the spleen, mostly related to T-cell function (200).

Individuals living in areas where malaria transmission is common develop immunity through repeated infection, and many become clinically immune by childbearing age (21, 196). However, women undergoing pregnancy for the first time are susceptible to infection by P. falciparum parasites expressing distinct variant surface antigens that enable them to be sequestered in the placenta (196). This is thought to be because chondroitin sulfate A (CSA) is expressed in the (placental) vasculature only during pregnancy, and so parasites that are able to bind to CSA do not have a selective advantage at any other time in life and have never been the target of the host immune response (19, 196). In pregnancy, parasites expressing a CSA-binding variant of PfEMP1 can be sequestered in the placenta, avoid splenic clearance, and establish infection in an otherwise partially immune individual, and several transcriptomic studies support the concept that there is specifically increased expression levels of *var2csa* in parasites isolated from the placenta (80, 201, 202).

Human gene expression in the placentas of malaria-infected women showed considerable perturbation, with distinct patterns related to both infection status and the presence or absence of histological inflammation (203). B-cell- and macrophage-related genes were particularly enriched, including *CXCL13*, a macrophage-derived B-cell chemokine. Transcriptomic findings, combined with additional reverse transcription-PCR (RT-PCR) and immunohistochemical analyses, led the authors of that study to speculate that macrophage CXCL13 drives B-cell recruitment to the placenta, antibody production, and further antibody-mediated activation of inflammation, in a pattern suggestive of lymphoid neogenesis (203). A subset of these differentially expressed immune response genes was negatively correlated with birth weight (203). This is a particularly important finding since low infant birth weight is a major risk factor for infant mortality (204).

###### (b) Parasite factors.

There are few studies examining parasite transcriptomes in association with the pathogenesis of noncerebral severe malaria. Dual RNA-seq revealed 126 parasite genes significantly associated with high fever, with the strongest association occurring with PF3D7_0500900 (serine/threonine protein kinase, FIKK family [FIKK5]) (25). However, those results should be interpreted with caution since fever spikes are thought to be related to parasite egress from RBCs, which is dependent on the distribution of the developmental stages of the parasites, which in turn is the greatest determinant of parasite gene expression. FIKK5, for example, shows substantial variation in expression across the developmental cycle *in vitro* (86). That same study identified 234 parasite genes associated with the proportion of whole-blood reads mapped to the parasite (a proxy for circulating parasite density), but these genes were poorly annotated at the functional level, making it difficult to understand how they may be related to the parasite load (25).

Comparison of parasite gene expression levels has been performed with a small number of adults with noncerebral severe malaria (predominantly subjects with renal impairment) versus those with uncomplicated malaria by using a custom microarray designed to identify a broader repertoire of genes from variant gene families of parasites (205). Given the small size of that study, it was surprising that 380 genes were identified as being differentially expressed, with a notable downregulation of genes associated with host cell entry in severe malaria as well as enrichment for metabolic processes and RNA splicing and the differential expression of a range of variant surface antigens (205). The generalizability of these findings remains to be determined.

In pregnancy-associated malaria, transcriptomic analyses of parasites from placental malaria have found modest numbers of differentially expressed genes other than *var2csa*, many of which are thought to be exported into erythrocytes but do not have well-defined functions (80, 201, 202). This suggests that beyond the importance of *var2csa*, additional mechanisms of the host-parasite interaction may also play a role in susceptibility to malaria in pregnancy.

###### (c) Host-pathogen interaction.

The association between host gene expression and parasite load is of interest because the parasite load is a determinant of severity and because restriction of the parasite load would indicate protection. Several studies of different patient groups have related host gene expression to parasitemia and found fairly consistent associations with the upregulation of neutrophil-, interferon-, phagocytosis-, complement-, and heme degradation-related genes (24, 25, 126). Assessment of the association of host gene expression with PfHRP2 concentrations, as a proxy for the total body parasite load, has not yet been done. The whole-body parasite load (which includes both circulating and sequestered parasites) is generally a much better predictor of outcome than circulating parasitemia (18, 26–30), and so this analysis will be important in future studies.

A direct correlation of host and parasite gene expressions was possible in only one study to date, which used dual RNA-seq to analyze whole blood from uncomplicated P. falciparum malaria cases in Indonesia (25). Host innate immune response genes were negatively correlated with parasite metabolic process genes, perhaps suggesting a direct impact of the host response on the restraint of parasite metabolism. However, most of the correlations between human and parasite gene expressions were positive, and among these genes, the most notable were human transcription factor genes positively correlated with parasite translational regulation (25). Despite caveats about a confounding effect of the parasite developmental stage in that study, it is intriguing to speculate that those observations may indicate a reciprocal regulation of fundamental biological processes between the host and parasite.

#### Biomarkers.

Identifying reliable biomarkers can improve diagnosis and the prediction of outcomes of infectious diseases. This is particularly important for malaria because while some individuals living in high-transmission settings will have parasitemia, which is not the cause of their illness, others will definitely have malaria but despite antimalarial treatment may develop severe disease, and others will have coinfections with malaria and bacterial pathogens, both of which require treatment (11, 27, 28, 132). Identifying these groups could stratify clinical care as well as studies of immunology, pathogenesis, and treatment. Biomarkers that can predict outcomes in animal models have the potential to improve the power of these models and to benefit the welfare of the animals by allowing accurate sample size calculations and the use of less-severe endpoints. Given the complexity of malaria pathogenesis, biomarker discovery may provide more than just a disease indicator but also greater insight into the cellular responses and physiology of the host and the pathogen. Increasingly rigorous methodology is now being applied to biomarker discovery, such as standards for reporting of diagnostic accuracy (STARD) (206), and transparent reporting of a multivariable prediction model for individual prognosis or diagnosis (TRIPOD) (207). Validation is required with multiple independent data sets along with standardized reporting of how well the biomarker predicts the outcome of interest. Most transcriptomic biomarker discovery studies of malaria have not met this standard of evidence; nonetheless, they suggest that robust biomarkers might be found in the future.

##### (i) Diagnosis of malaria.

Since we already have good tools to detect Plasmodium using microscopy, point-of-care antigen tests, and even PCR-based parasite detection (11), the utility of transcriptomics for the diagnosis of malaria may not be obvious. Gene expression signatures can be derived to distinguish diseases that have similar clinical presentations, such as distinguishing bacterial from viral infection (208) or tuberculosis from nontuberculous infection (209), demonstrating a proof of principle that each disease has a unique transcriptional signature that might form the basis of a rapid test to guide initial clinical management (210, 211). In a country where malaria is endemic, any such test would need to be able to distinguish malaria from other febrile illnesses, and so a gene expression signature of malaria would be essential. One of the first transcriptomic studies of malaria suggested that this would be feasible because unsupervised clustering was able to largely separate febrile malaria from other acute febrile illness (24). Clusters were particularly separated based on neutrophil-related genes and erythroid-related genes; however, modern variable selection methods and testing with an external validation data set would be needed to develop a sufficiently small, sensitive, and specific gene expression signature for diagnostic use.

##### (ii) Predictors of severity or death.

The majority of transcriptomic studies attempting to identify biomarkers of severe outcomes have been conducted with animal models rather than humans. In a comparison of gene expression patterns in mouse brains from four different strains, 31 genes were found to distinguish ECM susceptibility from resistance regardless of the mouse strain (174). The utility of gene expression biomarkers in brain tissue is limited, since this tissue cannot easily be sampled without killing mice, but it may provide a more refined experimental endpoint that does not require mice to develop end-stage ECM. Gene expression biomarkers in peripheral blood are arguably more useful, and putative examples have been identified from a comparison of the whole-blood transcriptomes of ECM-resistant and ECM-susceptible mice (173). Of a large number of differentially expressed genes detected by microarray analysis, a smaller number was confirmed by RT-PCR, and several of these genes (*c1qb*, *DnaJC15*, and *tk1*) were different enough between groups to be considered candidate biomarkers. *C1Q* was also identified as a putative biomarker of severe human malaria in a pilot study of 10 Malian children (171). Seven other candidate markers were all related to the immune response, including TLR genes.

Biomarker discovery for diagnosis or prognosis is not restricted to host gene expression only. Potential parasite biomarkers in humans have been investigated by transcriptomic profiling of parasites taken from children with CM, uncomplicated malaria, and asymptomatic malaria (212). Among them, only the comparison between CM and asymptomatic malaria found differentially expressed genes, 99 of which were upregulated and 135 of which were downregulated in CM. Further characterization of these genes revealed a large proportion of upregulated genes encoding Plasmodium exported proteins and variant surface antigen proteins, such as PfEMP1 and RIFIN, and downregulated genes included those encoding rhoptry-associated proteins, merozoite surface proteins, and Maurer's cleft two-transmembrane domain protein (212).

##### (iii) Markers of parasite drug resistance.

Resistance to antimicrobial agents, including antimalarial drugs, is a growing problem (213, 214). Widespread resistance could set back many of the gains made by malaria control initiatives over the last decade (11, 214). Artemisinin and its derivatives are the mainstay of antimalarial treatment globally, and emerging resistance is starting to spread from foci in Southeast Asia (11, 214, 215). Markers of artemisinin resistance were sought in a microarray-based transcriptomic study of 1,043 P. falciparum clinical isolates from 13 regions of Southeast Asia and Africa where malaria is endemic (216). The identification of genes significantly associated with the parasite clearance half-life strongly implicated the parasite unfolded protein response in mediating artemisinin resistance and also showed an association with reduced expression levels of DNA replication genes, presumptively linked to relative resistance in slower-developing early-blood-stage parasites.

## LEARNING FROM TRANSCRIPTOMIC STUDIES OF MALARIA

### Key Insights

#### The ups and downs of type I interferons.

The high frequency of AT-rich motifs in Plasmodium genomes, such as the ATTTTTAC motif in P. falciparum, is implicated in the induction of type I interferons (127). The role of type I interferons in malaria has been enigmatic because of conflicting data from animal models indicating roles in both protection and pathology (127, 142, 199, 217, 218). Emerging findings suggest that the timing, source, and regulation of type I interferons are crucial (219), and it is interesting to note that this might have been inferred earlier from data in the transcriptomic literature. In humans and mice, type I interferon-related genes are consistently induced in blood early in infection (in uncomplicated disease) (126, 127, 129, 173), but relative downregulation is seen in later, more severe infection or with a higher parasite load (129, 173, 187) (Fig. 3). Interestingly, this type I interferon signature is found in PBMCs (127), whole blood (129, 173), and isolated neutrophils (220).

In contrast, increases in type I interferon-related gene expression levels in brain are temporally associated with the onset of ECM (69, 144, 174, 175, 178, 179), whereas higher early expression levels in spleen are associated with a better control of parasitemia (199), and sustained high expression levels in spleen are associated with ECM (217). This fits very well with the evolving paradigm, developed through functional studies, that an early burst of type I interferons may be necessary to enhance innate and adaptive responses, while sustained production may suppress pathological immune responses but risks compromising the control of the parasite load (218, 219). Thus, the timing of the up- and downregulation of type I interferon signaling determines a fine balance between immunopathology and parasite survival, and influencing this balance through the kinetics of AT-rich DNA release may have evolved as a parasite strategy to manipulate the host response (142, 218).

#### Pathological effects of neutrophils.

Neutrophils are one of the most numerous leukocyte populations in blood, yet there has been disproportionately little investigation of their role in malaria. Higher neutrophil-associated gene expression levels are a consistent feature of analyses of whole-blood transcriptomes from uncomplicated and severe human disease (24, 126, 187). In contrast, the sparse data from rodent models do not show an induction of a neutrophil-associated gene expression signature in whole blood in ECM (173) but show an association with lung pathology (137). The simplest explanation for these differences might result from changing proportions of neutrophils in blood.

Relative neutrophilia is frequently described in human cases of malaria (221, 222), particularly in association with severe disease and high-level parasitemia, whereas this is not always the case in rodent malaria models. However, differences in neutrophil counts are not the only explanation. In cases of retinopathy-positive CM, neutrophil gene expression signatures were greatly increased in comparison to those in cases of retinopathy-negative CM despite very similar neutrophil counts (187). Another explanation may be genuine transcriptional differences between whole-blood neutrophil populations, possibly arising from the mobilization of immature neutrophils from the bone marrow. This may explain the particular enrichment of genes encoding neutrophil granule proteins, being regulated during neutrophil development and particularly expressed in immature neutrophils (223).

Consistent with this, immature neutrophils and elevated circulating levels of their granule proteins are detected in cases of severe and uncomplicated malaria (221, 224). Many neutrophil granule proteins can be damaging to host tissues, and it is tempting to speculate that this indicates a pathogenic mechanism in humans. Female C57BL/6 mice, which are commonly used in malaria research, have among the lowest circulating neutrophil counts of all mouse strains (225). Although neutrophil mobilization from bone marrow has been described for rodent malaria (226, 227), neutrophil counts in peripheral blood can go up or down (226–228), possibly reflecting the rate of egress from bone marrow, trafficking to other organs, and cell death.

Proving a functional role for neutrophils in rodent model pathogenesis, and extrapolating the data to human disease, is also challenging. Antibody depletion of neutrophils has been used to assess their role, but common antibodies used for this purpose can also deplete monocyte subpopulations (229, 230). Despite this, additional evidence supports the roles of neutrophils in malaria-induced lung and liver injury (220, 228) and of a neutrophil subpopulation in ECM (227). The prominence of neutrophil-related gene expression in human cases of malaria clearly indicates that more work is needed to understand exactly what these cells contribute to both defense and disease.

#### Variations in parasite gene expression *in vivo*.

The broad similarity of *in vivo* and *in vitro* parasite transcriptomes, particularly after accounting for the parasite developmental stage, is discussed above. However, the amount of variation from the *in vitro* “standard” that occurs *in vivo* remains to be fully quantified, as do the implications of any *in vivo* variation. Differences in parasite gene expression detected between different *in vivo* situations (117, 216), including those under antimalarial drug pressure, suggest that the *in vivo* variation can be both substantial and responsive to the within-host environment, even if, on average, it is similar to the *in vitro* situation. The implications of such *in vivo* variation may be very important. The use of laboratory-adapted parasites for high-throughput drug and antibody screens may either overestimate or miss *in vivo* effectiveness. Unfortunately, the factors that drive *in vivo* variations are currently poorly defined, but their characterization might allow the adaptation of culture conditions to recreate elements of *in vivo* variability.

### Closing Gaps in Knowledge: General Principles

#### How similar are animal models and human disease?

Although it is clear that there are differences between severe malaria in animal models and human disease (172, 180, 197), there is no agreed way to quantify the importance of the differences or similarities for understanding pathogenesis. This is not unique to malaria research; similar controversies arise for other infectious diseases, such as tuberculosis (231), meningococcal disease (232), and typhoid (233). One way to objectively identify similarities would be through systematic transcriptomic comparisons between species, across organs, at different time points, and over the spectrum of severity (234). The easiest comparisons in practice will be comparisons of blood transcriptomes between humans and rodent models, as has been tried for tuberculosis (231), but comparisons with other tissues will also be needed for a complete understanding (234). In this way, the components of a model that have relevance to human disease may be identified and studied further, even if other aspects of the model are different. Studies with outbred, wild-derived, or true-wild mice may also be highly informative because the greater genetic variation between these mice (235) may increase variations in gene expression associated with variations in the parasite load, severity of anemia, or organ dysfunction at the same time point during infection, recapitulating the variability found in humans.

#### Variation in pathogen genomes.

The involvement of the highly polymorphic *var*, *rif*, and *stevor* multigene families in the pathogenesis of severe malaria is attracting increasing interest (19, 236), but accurately determining the expression levels of these and other highly polymorphic genes in global gene expression analyses remains challenging because of current reliance on a reference genome for the quantification of transcript levels. For bacterial pathogens, a similar challenge can arise through different mechanisms, with the core genome potentially being supplemented by a flexible genome of additional genes present at various frequencies (237). The flexible genes may be acquired or lost by mutation and selection during the course of population growth and by the transfer of genetic material between bacterial species. One strategy for improving quantification is a targeted approach sequencing just the most abundant variant genes, as was recently applied to the P. falciparum *var* transcriptome (192). A more comprehensive strategy would be complete genome and transcriptome sequencing on the same pathogen isolates. Assembly would be facilitated by longer sequenced reads using, for example, MinION and PacBio sequencing technologies (238, 239). Cost remains an issue for this approach, particularly because it would be important to collect as many clinical isolates as possible to obtain a complete picture of their complexity and rigorously define associations with severity.

#### Are we sampling the right tissues?

The most useful transcriptomic information about the host response or host-pathogen interactions will likely be derived from RNA extracted from specific tissues rather than bulk sampling of tissues. For example, analysis of whole brain or whole spleen, composed of many cell types with differing functions, may misrepresent specific interactions involved in protection or pathogenesis. Ideally, we would like to investigate much more specific *in vivo* interactions in malaria (Fig. 1), for example, mosquito saliva, sporozoites, and skin; Kupffer cells, hepatocytes, and sporozoites; hepatocytes, tissue schizonts, and CD8 lymphocytes; and brain endothelial cells and sequestered parasites. Techniques are evolving for the microscopic dissection and capture of specific cells from tissues and for the purification of single cells from blood and organs (36, 240) (Fig. 2). The combination of these techniques with RNA-seq protocols suitable for histological specimens and low-quantity RNA now opens the door to interrogating these interactions in great detail (240).

#### Which clinical phenotypes should be studied?

Future studies need to give careful consideration to the information that might be gained from comparisons of different clinical phenotypes. To better understand the pathogenesis of severe malaria, there is a clear need for a dual RNA-seq analysis comparing cases of severe and uncomplicated malaria. Deeper insights may be gained from comparisons of subjects with discrete phenotypes of severe malaria, such as severe anemia, acidosis/hyperlactatemia, and CM. Comparisons between subjects with malaria and subjects with asymptomatic infection, matched for parasite loads, may be particularly helpful to understand the nature of antidisease immunity.

#### Are we looking at the right stage of infection?

Looking at events preceding the onset of severe disease due to infection is necessary to understand the underlying pathogenic mechanisms, but targeting these mechanisms when a patient presents with severe disease may be ineffective because the damage has already been done. There are notably no studies of malaria examining gene expression profiles associated with recovery from severe disease, during the first few days after the initiation of antimalarial treatment, and such studies may be much more informative for the identification of targets for adjunctive therapies to improve outcomes of malaria and other infectious diseases.

#### Association and causation.

Within transcriptomic data sets, it is inevitable that the expressions of many genes will be highly correlated with each other and with outcomes of interest, and this makes it particularly difficult to separate association from causation in observational studies. We believe that there are approaches that might enhance the identification of causal relationships. One relatively simple approach is to look at differences between different biological phenotypes of the outcome of interest. For example, comparisons of uncomplicated malaria with each of the different phenotypes of severe malaria (hyperlactatemia, CM, and severe anemia) may reveal gene expression common to all severe phenotypes and gene expression specific to each one. Since the severe phenotypes share many common risk factors, such as parasite load (13), we would expect the genes specific for each phenotype to be more likely to have causal relationships. Similarly, quantitative or dose-response relationships can be used to identify those genes that are highly correlated with specific features, such as the platelet count or lactate concentration.

Even stronger evidence may come from one of the most powerful epidemiological methods for demonstrating causation, Mendelian randomization (241). This epidemiological technique uses natural population variation in a gene with a known function to examine the causal effect of exposure, usually requiring a large set of genotyped samples. A seminal example is the use of the protective effect of sickle-cell trait against developing malaria to demonstrate that malaria causes susceptibility to bacterial infections in humans (242). A similar approach might be employed to estimate the proportion of gene expression that is responsible for limiting the parasite load rather than occurring solely as a consequence of the parasite load. Sickle-cell trait or other red cell polymorphisms that limit parasite growth independent of the host immune response might be used as the instrumental variable.

Regardless of the approach, new hypotheses may be generated based on gene expression data, which can be tested by using reductionist approaches *in vitro* or in animal models for experimental validation. In humans, causality can be assessed directly in interventional studies, with transcriptomics providing a means to elucidate the mechanism of action of new treatments in clinical trials.

### Lessons for Study Design and Analysis

It is clear that many studies on malaria had suboptimal elements in their design. Principles for improving the design of malaria studies will also apply to many other infectious diseases (Table 2). The overarching principle is that if a study is not designed to answer the research question, no novel statistical approach and no amount of data mining are likely to be able to answer the question. Community guidelines for both microarray and RNA-seq reporting already exist and will likely evolve to keep track with increasingly complex analytical approaches (243, 244).

**TABLE 2:** Lessons for the design of infectious disease transcriptomic studies

Phase	Challenge	Problem	Example	Solutions
Design	Defining study objective	*Post hoc* specification of objectives invalidates statistical analyses	Use of principal-component analysis plots to identify groups that differ the most and then selection of these groups for further analysis	Prespecify objectives; prespecify hypothesis; prespecify analysis plan
	Bias	Systematic differences between comparison groups in sample selection, data availability, assessment, or analysis; cannot be eliminated statistically	Selection of control subjects from a different country to cases	Select all samples from the same representative population; perform blind assessment of samples; perform blind data analysis
	Confounding	Factors associated with the outcome of interest may influence the association between gene expression and outcome	Likelihood of symptomatic malaria or asymptomatic parasitemia is influenced by age, prior exposure, and parasite load	Collect data on known confounders to allow statistical adjustment; match for known confounders; perform within-subject comparisons to eliminate unknown confounders
	Generalizability	Statistically significant results can be obtained from small sample sizes but may not be representative of the population of interest	Comparison of groups of 3 inbred mice may be representative of infection in those mice, but data from similar-sized studies with humans almost certainly will not be representative	Use sufficient biological replicates to capture the diversity of the population; replicate findings with independent samples; replicate findings with alternative methods; replicate findings in independent population(s)/exptl models
	Causal inference	Observational studies cannot prove that variation in gene expression causes the outcome of interest, but evidence for a causal association may be strengthened by certain features of study design	Higher type I interferon response gene expression levels in uncomplicated malaria than in severe malaria (Is it protective?)	Compare graded outcomes for gene expression “dose-response” relationships; use Mendelian randomization designs; assess during exptl study or interventional clinical trial
Sampling	Isolation of host and pathogen RNAs	Both species may be present in the sample, but the ratio of host/pathogen RNA can determine success of the expt	Human and Plasmodium transcriptomes are easily assessed in blood of patients with high-level parasitemia, but the Plasmodium transcriptome is difficult to assess when the level of parasitemia is low	Estimate likely amounts of host and pathogen RNAs to assess feasibility prior to sampling; consider focused sampling to increase the ratio of pathogen/host cells; consider enrichment of pathogen RNA
Analysis	Cellular heterogeneity	Differences in cellular compositions of samples can dominate transcriptional profiles	Infection often changes proportions of different leukocyte populations in blood	Apply deconvolution algorithm to adjust for differences in cell mixture in bulk transcriptome; use flow cytometry analysis to directly determine cellular composition; purify cell types of interest before transcriptome analysis; use single-cell RNA sequencing
	Adjustment for confounders	Statistical adjustment is needed if matching for confounders cannot be achieved	Adjustment for parasite load in comparison of cases of severe and uncomplicated malaria	Logistic and linear regression models can be implemented in many transcriptomic analysis tools
	Discovery of optimal biomarkers	Expression of many genes may be associated with outcome, but it is difficult to select the smallest combination with the best out-of-sample prediction	Which combination of transcripts best predicts clinical deterioration in a child with uncomplicated malaria	Apply variable selection algorithms to a training data set; confirm with a separate test data set; validate with at least 1 external data set
Reporting	Maximize reuse of data	Provision of metadata allows maximum future reuse of transcriptomic data	Complete subject- or sample-level data allow secondary analyses to be performed	Observe community standards for reporting; make metadata publicly available

## TRANSCRIPTOMICS IN FUTURE HOST-PATHOGEN RESEARCH

Fifteen years of transcriptomic studies of malaria have brought many insights but also highlighted the complexity and challenges of studying host-pathogen interactions in this way. For other infectious diseases, some of these challenges may be even greater. In the next decade, advances in transcriptomics will likely transform our understanding of host-pathogen interactions in many infections. Specific examples might include unraveling the triggers and consequences of bacterial toxin and protease production in virulent staphylococcal and streptococcal infections; establishing the mechanisms underlying clearance, latency, or progression in tuberculosis; and defining protective and harmful host-pathogen interactions in emerging infections, such as Ebola and Zika viruses.

This will be facilitated by technical advances, such as long-read sequencing technologies and single-cell sequencing, that will allow unprecedented levels of detail to be described. The greatest challenge may become the synthesis of the huge amounts of data and detail into a model that is comprehensible to the human mind.

We believe that important insights will come from the integration of multiple layers of data, such as transcriptomic, genomic, proteomic, and metabolomic data (1), combined with comprehensive descriptions of clinical and other pathophysiological features of infections. This will need to be done in increasingly large (and therefore costly) studies, because the more layers of data that are integrated, the greater the dimensionality. Methods for effectively reducing this dimensionality with minimal compromise in discrimination will no doubt evolve in parallel. Usefulness and generalizability will need to be maximized by the collection of consistent types of data between studies so that their combination and meta-analyses can be performed.

To ensure the translation of discovery to clinical application, researchers will need to better characterize the clinically relevant components of their model systems and achieve optimal sampling of tissues with the most-relevant host-pathogen interactions. This will require collaboration across disciplines, bringing transcriptomics together with advances in imaging, minimally invasive sampling, microscopy, and viable-cell separation. Studies with humans will be greatly enhanced by embedding within formal epidemiological studies with rigorous design principles. Clinical trials of antimicrobials and adjunctive therapies represent rare opportunities where humans are subjected to experimental manipulation, and collecting samples for transcriptomic analyses will be helpful for mechanistic interpretations of their results.

Mathematical developments will likely facilitate new approaches to studying trajectories of infectious diseases, overcoming the inherent limitation that naturally infected human subjects present only a snapshot view of the dynamic process of infection (136). Approaches have already been developed for fate mapping of single cells based on transcriptional similarities over a developmental continuum (245), and a similar approach may be taken to the course of human disease where the transcriptome of an individual at a single point in time might be mapped onto a disease trajectory from asymptomatic to mild to severe, by reference to the transcriptomes of each disease state.

Transcriptomic studies fairly consistently show that different gene expression signatures characterize infections with different pathogens and different stages of infection with the same pathogens (210, 211). This means that there is a high likelihood that various selection methods applied to transcriptome analyses in any infectious disease will reveal small sets of transcripts as diagnostic or prognostic biomarkers. This has the potential to revolutionize many aspects of clinical infectious disease practice, if these could be turned into rapid tests administered at the point of care. Currently, commercial methods to allow these RNA signatures to be used as near-patient tests do not exist (211), but as the potential impact of and demand for such methodologies are realized, it seems inevitable that the technological problems will be solved.

In conclusion, we are optimistic about the advances in the understanding of host-pathogen interactions that will come from future transcriptomic studies and the benefits that may come from these studies if they are well conducted. We predict that the results of these studies will force researchers to consider much more dynamic models of infection, where changes in the host response may affect pathogen behavior and vice versa, and that the simultaneous study of host and pathogen will increasingly be viewed as being essential.
